# Nanomaterial-Based Approaches for Neural Regeneration

**DOI:** 10.3390/pharmaceutics11060266

**Published:** 2019-06-08

**Authors:** Raluca Ioana Teleanu, Oana Gherasim, Tudor George Gherasim, Valentina Grumezescu, Alexandru Mihai Grumezescu, Daniel Mihai Teleanu

**Affiliations:** 1 “Victor Gomoiu” Clinical Children’s Hospital, “Carol Davila” University of Medicine and Pharmacy, 050474 Bucharest, Romania; raluca.teleanu@umfcd.ro; 2 Department of Science and Engineering of Oxide Materials and Nanomaterials, Faculty of Applied Chemistry and Materials Science, Politehnica University of Bucharest, 011061 Bucharest, Romania; oana.fufa@gmail.com; 3 Lasers Department, National Institute for Lasers, Plasma and Radiation Physics, 077125 Magurele, Romania; valentina_grumezescu@yahoo.com; 4 National Institute of Neurology and Neurovascular Diseases, 077160 Bucharest, Romania; tudor.gherasim@gmail.com; 5 Emergency University Hospital, “Carol Davila” University of Medicine and Pharmacy, 050474 Bucharest, Romania; daniel.teleanu@umfcd.ro

**Keywords:** nervous system injury, nanomaterial, neuroregeneration

## Abstract

Mechanical, thermal, chemical, or ischemic injury of the central or peripheral nervous system results in neuron loss, neurite damage, and/or neuronal dysfunction, almost always accompanied by sensorimotor impairment which alters the patient’s life quality. The regenerative strategies for the injured nervous system are currently limited and mainly allow partial functional recovery, so it is necessary to develop new and effective approaches for nervous tissue regenerative therapy. Nanomaterials based on inorganic or organic and composite or hybrid compounds with tunable physicochemical properties and functionality proved beneficial for the transport and delivery/release of various neuroregenerative-relevant biomolecules or cells. Within the following paragraphs, we will emphasize that nanomaterial-based strategies (including nanosized and nanostructured biomaterials) represent a promising alternative towards repairing and regenerating the injured nervous system.

## 1. Introduction

The main role of a nervous system is to integrate the corresponding organism into the world around it, by balancing a very precise relationship between its fundamental principles, namely data collection, information processing, and response selection. Asserting the nature of neuronal networks, the accuracy and speed by which billions of neurons come together within the central nervous system to achieve different functions is astonishing. Some authors have gone as far as to consider that this level of complexity is an expression of quantum mechanics, thus giving life to the quantum brain and quantum conscience concepts [1,2].

In any case, although the nervous tissue is such a vital component for invertebrate and vertebrate organisms, with regards to the human nervous system, the adult neuron is a very fragile cell, being irreplaceable within the central nervous system (except for a few suspected situations). The majority of a neuron’s energy reserves are used for maintaining synaptic potentials and neurofilament structural dynamics, which form the main backbone of neuronal computations. For this reason, other supporting cells (neuroglia) are required to help with vital key roles, such as ion and water homeostasis, defense against oxidative stress, energy storages, tissue repair and scar formation, modulation of synaptic activity through gliotransmitters, and synapse formation/ remodeling [3,4]. Since the main focus of the current paper is directed towards medical end-goals, any discussion moving forward will be considered with regards to human physiology.

As we previously mentioned about the exception to the irreversible neuronal damage concept, it must be stated that there is conflicting evidence for neurogenesis within the central nervous system (CNS), with supporting studies pointing mainly at the sub-granular zone of the hippocampal dentate gyrus, subventricular zone of the lateral ventricular wall and the amygdala [5], where learning and memory processing is considered to unfold, while other studies being unable to identify such properties in the adult mammalian brain [6].

In any case, outside of these territories, any injury to the CNS that is severe enough to produce neuronal cell body death will be followed by a cascade of biochemical events that ultimately lead to liquefactive necrosis. The necrotic core will be surrounded and isolated by a glial scar, in time ultimately forming a cerebrospinal fluid-like low-density well-circumscribed region that replaces the once noble neuronal tissue. The end result will be the subsequent loss of function and expression of different types of clinical syndromes [7,8]. With this in mind, it is important to understand that direct neuronal cell body is not the only cause of clinical dysfunction, since white matter lesions, hence axonal injury, can also result in severe dysfunction, by interrupting important signals from the neuronal cell body [9,10]. It is also important to note that any lesion, be it grey or white matter located, will also damage surrounding neuroglia, which are represented by astrocytes, oligodendrocytes, ependymal cells and microglia in the CNS, and Schwann and satellite cells in the peripheral nervous system (PNS).

In contrast, the peripheral nervous system’s auto-repair capacity greatly outperforms that of the CNS, being capable of regenerating nerve fibers with speeds ranging from 1 mm/day in small nerves to 5 mm/day in large nerves [11]. The most important factor influencing the regeneration potential of the two systems is the type of glial cell present and its particular behavior in response to threat, injury, and maintenance.

Schwann cells wrap around only one axon, thus multiple cells are required to fully insulate a nerve fiber. When nerve damage occurs, the distal section of the axon (relative to the site of injury) remains electrically excitable, but will eventually undergo Wallerian degeneration within 24–36 h of the lesion. This phenomenon refers to the process of cleaning the myelin sheath fragments and axonal debris that follows the axonal skeleton and membrane disintegration, which occurs due to the severing of the molecular transport pathway from the neuronal cell body. As it currently stands, Schwann cells, alongside recruited macrophages, are implicated in this process and it ends with the former proliferating and forming special tubes (Büngner bands) in which they express surface molecules that guide the axonal growth cone of the proximal regenerating fibers [12,13].

In comparison, oligodentrocytes can produce myelin sheaths for up to 50 axons, but their survival depends on signals from within the axons themselves [14]. This means that during the Wallerian degeneration that follows white matter lesions, the axonal disintegration will eventually induce apoptosis of the corresponding oligodendrocytes, thus failing to quickly recruit macrophages to help clean the myelin/axonal debris, which by themselves have inhibitory effects for the regenerating axonal growth cones, alongside the newly forming glial scar that also hinders reinnervation. Microglia remains the primary cellular detritus cleaner, but they tend to hypertrophy, instead of transforming into fully operational phagocytic cells, which makes them slower in comparison to macrophages (which eventually enter the lesion site after 3–4 days) [15].

Fortunately, although the CNS lacks the cellular regenerative capacity of the PNS, it compensates to a certain degree of functional recovery through a different and highly remarkable mechanism, known as neuroplasticity [16,17]. This biological property emerges from the sheer electrical complexity of neuronal networks linked throughout the CNS and their dynamic activity-modulated synaptic connection maintenance, in accordance with Hebb’s postulate: “neurons that fire together, wire together”.

In other words, through positive behavioral modifications reinforced by neuro-rehabilitation techniques, it is possible to recruit the help of surviving adjacent neurons and the corresponding intact white matter tracts (projection, association and commissural fibers) that connect them to ipsilateral or contralateral neuronal populations within the CNS. The new synaptic connections formed at various levels either try to relocate lost cortical functions to other adjacent areas of the ipsilateral neocortex, or try to compensate and reroute signals in an attempt to “borrow” neuronal functions from the contralateral cerebral hemisphere (or spinal cord) and bypass the dysfunctional regions and/or connections [18,19].

## 2. Nervous System Regenerative Therapy

The intrinsic ability of healthy nervous system to remodel itself, to generate particular experience-derived response and to analyze complex information is impressive. Injured central and peripheral nervous system leads to permanent tissue damage and significant functional impairment. The limited innate ability of neural tissue for self-regeneration represents an attractive and challenging concern for scientists regarding the development of novel neuroregenerative strategies. For what concerns the particular field of modern neural tissue bioengineering, the regenerative potential related to nanostructured biomaterials have been assessed with respect to traumatic or degenerative lesions occurring in central or peripheral conditions.

The clinical and experimental therapeutic strategies for traumatic CNS (including brain and spinal cord injury) are inherently limited due to (i) cellular heterogeneity and complex synaptic connections; (ii) presence of endogenous inhibitory molecules associated with myelin and extracellular matrix (ECM) or inhibitory proteins secreted by glial scar’s cells, which generally prevent axonal outgrowth and regeneration; and (iii) complex ultrastructure of blood-brain barrier (BBB), which provides the intrinsic regulation in CNS-related physiological and pathophysiological events [20,21,22,23,24].

For what concerns the first limitation in CNS regeneration, a massive progress was reported by means of stem cells—based therapy. Thanks to their impressive potential to differentiate into mature and functional neurons and neural cells, stem cells proved excellent and clinically effective candidates for remyelination, axonal outgrowth and generation of signaling pathways [25,26,27]. Important results were observed in CNS therapy by considering pluripotent human-derived embryonic stem cells [28,29,30,31], fetal stem cells [32,33] and adult stem cells [34,35,36]. Moreover, other promising pre-clinical results for CNS repair were mentioned by inducing transdifferentiation of resident and mature cells into neural cells [37,38,39].

With respect to the hostile microenvironment of the lesion site within the CNS, modern therapeutic approaches enable the subtle and precise alteration of injury site, as to combat inhibitory effects and encourage regenerative processes [21,24,40]. By gathering extensive molecular and cellular biology knowledge, as well as biotechnology and bioengineering expertise, various biomolecules were assessed as beneficial for normal neural development and functional regulation, including growth factors [41,42,43,44], neurotrophins [45,46] and ECM proteins [47,48,49,50].

In addition to the multidisciplinary considerations mentioned in the last paragraph, research in the field of pharmacology remains essential in overcoming the BBB for CNS regenerative applications. By properly selecting and specifically tuning the therapeutic candidate, various surpassing routes can be achieved, such as BBB permeabilization followed by infiltration, endothelial-mediated transcytosis and endothelial endocytosis followed by abluminal exocytosis [40,51,52,53]. Given the intricate ultrastructure and accurate function of cerebral capillaries, surpassing the BBB is a major challenge in neuropharmacology [54,55]. The implication of BBB in managing CNS conditions is essential and extensive, but does not represent the main topic of the present paper. For thorough understanding, systematic data evidence that specific and complex pharmaceutical considerations and solid research are required to successfully deliver bioactive molecules across BBB [56,57,58,59,60,61].

By contrast, the PNS fibers possess significant self-healing ability, mainly thanks to the constructive role of Schwann cells, which represent the counterparts of CNS glial cells [51,62].

When the injury creates small gaps in the peripheral nerves (corresponding to maximum axonal transection of 5 mm), direct anastomosis occurs and spontaneous regulatory processes mediated by local biomolecules contribute to fascicular coaptation, axonal repair and nerve reinnervation [63,64,65].

On the other hand, the proper repair and regeneration of PNS nerves with larger gaps require the implantation of nerve grafts [66,67,68]. The gold standard therapeutic procedure used for severely injured peripheral nerves relies on autologous nerve grafts, which particularly extinguish immunogenicity issues and favors microstructural repair and functional recovery [66,69,70]. Impressive neuroregenerative potential was also observed for biomodified acellular nerve allografts [71,72,73]. Furthermore, tremendous attention was directed towards clinically relevant artificial grafts, namely nerve guidance conduits that exhibit outstanding capacity to regenerate the injured peripheral nerves [74,75,76]. Herein, the current approaches consider acellular grafts (including membranes, meshes, scaffolds, tubes and gels) derived from ECM components [77,78,79,80,81] or new biomaterial-based architectures [82,83,84,85,86].

The nanotechnology-derived techniques to manipulate matter at atomic and molecular scale enable impressive methodologies and approaches to synthesize and engineer novel biomaterials. Thanks to their versatile functionality and tunable biological behavior, nanomaterials proved suitable candidates for neural repair and regeneration, being efficiently involved in modifying the nervous tissue microenvironment [87,88,89], the regulatory process of neural responses [90,91,92], the specific and selective delivery strategy of relevant drugs [93,94,95] and biomolecules [96,97,98].

## 3. Nanomaterials for Central Nervous System Regenerative Applications

Paclitaxel-encapsulated liposomes loaded within collagen microchannel scaffolds were recently proposed as a promising strategy for spinal cord injury repair. Following the treatment with liposomes embedded with the microtubule-stabilizing chemotherapeutic agent, an enhanced neuronal differentiation of neural stem cells (NSCs) was reported. The microchannel scaffolds provided a sustained release of paclitaxel from the liposomes, which resulted in an enhanced differentiation of both grafted and endogenous NSCs into mature and functional sensory and motor neurons (as assessed in a rat animal model with complete thoracic transection). Moreover, the collagen scaffolds loaded with paclitaxel-encapsulated liposomes promoted the animal locomotion recovery, as evaluated for up to 8 weeks [99].

The incorporation of growth factors (GFs) within nanomaterials and nanodevices (either by physical or chemical loading) represents a suitable and versatile approach adopted for neuroregenerative or neuroprotective strategies designed for peripheral or central neural therapies [65,100]. Dextrin-GFs conjugates were recently evaluated as beneficial platforms for local and sustained delivery of bioactive proteins involved in neural regenerative processes, providing enhanced and prolonged proliferation, specific nerve cell differentiation and inhibited apoptosis of mouse-derived neural stem cells (mNSCs). The biopolymer-protein formulations, obtained by conjugating succinoylated dextrin with epidermal growth factor (EGF) and basic fibroblast growth factor (bFGF), proved a sustained release of GFs following the incubation with amylase at concentrations similar to the cerebrospinal fluid physiological levels. As assessed in mNSCs cultures, dextrin-GF conjugates stimulated the expression of nestin protein (associated with neural progenitor), as well as the regulated expression of neural differentiation markers (associated with neuron, astrocyte and oligodendrocyte cells) [101].

Thanks to their intrinsic ability to modulate immune response by specifically binding the inflammatory monocyte receptors, synthetic and highly negatively charged nanoparticles—also known as immune-modifying nanoparticles (IMPs)—are in the spotlight of modern tolerant immune nanoparticle-based pharmacotherapy [102,103]. The intravenous administration of commercial IMPs based on carboxylated poly(lactide-co-glycolide) (PLGA, 500 nm particle size) in mice with spinal cord injury resulted in immediate reduction of the local inflammatory response, but also in the long-term reduction of fibrotic scarring and the levels of chondroitin sulphate proteoglycans deposits (as assessed for up to 10 months). Moreover, the PLGA-based treatment also maintained glial scarring and demyelinization levels, increased regional and caudal axon densities (as evaluated for up to 6 months) and significantly improved motor function [104].

In a study performed by Zamproni and co-workers, spherical micro- and nanoparticles based on PLGA were evaluated as promising encapsulation and release platforms for stromal cell-derived factor 1 (SDF-1), a chemokine responsible for neuron migration during embryonic development [105,106]. The obtained systems (with 4.83 ± 0.33 µm and 167.9 ± 0.38 nm mean particle size, respectively) enabled superior encapsulation of SDF-1 (above 84%) and resulted in effective and sustained release of the protein, with a particular prolonged release profile in the case of the PLGA nanoparticles. Both polymeric formulations enabled physiological levels of the released SDF-1, thus providing the suitable microenvironment for cellular chemotactic response. The PLGA/SDF-1 system proved neurogenesis potential only in nanoparticle formulation, since the investigated traumatic mouse brain injury was significantly repaired by the superior recruitment of neuroblasts towards the injured site [107].

Another study proved the efficiency of negatively charged PLGA nanoparticles (293 ± 19 nm) to provide the encapsulation-free release of positively charged CNS-relevant molecules from composite polysaccharide hydrogels by means of sole short-range electrostatic interactions. The copolymer nanosystems loaded with SDF or NT-3 (neurotrophin-3) and BDNF (brain-derived neurotrophic factor) molecules by electrostatic adsorption, resulted in a burst-free and sustained release (for up to 28 days) of the CNS proteins from hydrogels, which are based on cross-linked methylcellulose (XMC) or physically blended hyaluronic acid and methylcellulose (HAMC), respectively. Interesting fact, similar release profiles were reported in the case of protein-embedded and non-covalently protein-loaded PLGA nanoparticles, with only an increased amount of released proteins being reported in the first case [108].

The same research group observed the beneficial release of SDF chemokine and chondroitinase ABC (ChABC, an enzyme purified from *Proteus vulgaris* that is responsible for the degradation of chondroitin sulphate proteoglycan within glial scars, with inhibitory effects against myelin proteins within the nervous system) [109,110] with respect to the combined regenerative potential of the neural tissue. As observed in a rat model for up to 8 weeks, the thoracic local injection of methylcellulose hydrogels obtained by cross-linking either with SDF-loaded PLGA nanoparticles or with ChABC specific peptide and protein, resulted in improved and sustained locomotor behavior. When compared to sole chemokine-loaded hydrogels, both ChABC-loaded XMC and XMC co-loaded with ChABC and SDF enabled reduced levels of glial scars’ specific glycosaminoglycan chains and enhanced the migration of endogenous neural precursor cells [111].

A multi-step synthesis process was employed for the formation of complex nanosystems based on chitosan and branched polyethyleneimine (CS-g-PEI), conjugated by means of polyethylene glycol (PEG) with covalently linked cyclic arginine-glycine-aspartate (cRGD) and twin-arginine translocation (TAT) peptides. The resulted positively charged and spherical shaped polyplexes (mean particle size below 150 nm) proved excellent biocompatibility and DNA condensation capacity (as evaluated by monitoring a fluorescent plasmid reporter gene) in NIH 3T3 and 293T normal cells and HeLa tumoral cells. Moreover, improved transfection potential was assessed in all cell cultures, and the 293T cells treated with polymeric carriers embedded with NT-3 gene resulted in neuron and astrocyte differentiation, and neurite growth stimulation in neural stem cells derived from mice. Within this study, the complex polymeric carriers embedded with the gene encoding the NT-3 protein—a representative of nerve growth factors (NGF) which promotes peripheral or central neural survival, differentiation and new synapses formation [112,113]—were evaluated as a promising stem cells-based strategy for neural therapy [114].

An attractive strategy embraced to develop neuro-active platforms intended to provide enhanced cellular recruitment, sustained survival, and retention of cells, is to induce or potentiate the sensitivity of biomaterials for specific receptors involved in chemokine-mediated regenerative processes [115,116]. For example, biomaterials based on hyaluronic acid HA proved significant roles towards the overexpression of chemokine receptors in different stem cells types [117,118].

Porous composite hydrogels based on hyaluronic acid and laminin (HA-Lm) demonstrated stiffness values similar to native neural tissue and suitable microstructure for cellular migration, which resulted in enhanced survival and proliferation of neural progenitor stem cells (NPSCs). The composite hydrogels stimulated the overexpression of CXCR4 receptor (the principal receptor of SDF-1α chemokine), which subsequently promoted the chemotactic-guided migration of NPSCs by following SDF-1 gradients [119]. Furthermore, the co-injection of NPSCs suspension and HA-Lm gels into adult mice brain significantly increased cellular transplant retention during a 3-day treatment. Also, an increased and sustained migration of transplanted NPSCs towards the injection site of exogenous SDF-1α was reported. Complementary results proved that the enhanced transplant retention was a consequence of sole HA-Lm composite gel, whereas the in vivo migration of transplanted NPSCs was the consequence of SDF-1α-mediated chemotaxis [120].

Jian and co-workers recently assessed the neural regenerative potential of hydrogel matrices based on HA incorporated with polyelectrolyte complex nanoparticles (PCN) based on heparan sulphate or chondroitin sulphate non-covalently loaded with SDF-1α chemokine or bFGF growth factor, respectively. As evaluated during in vitro tests, the nanostructured hybrid materials proved an extended preservation time for the loaded bioactive molecules and showed sustained release, but also manifested an accelerated biomolecule release, following the exposure to matrix metalloproteinase (MMP, zinc-containing and calcium-dependent proteolytic enzymes up-regulated after brain injuries that are capable of degrading all components of ECM during structural and functional tissue repair) [121,122,123]. Moreover, the biomolecule-incorporated PCN, loaded within HA matrix, proved the ability to recruit endogenous NSC by chemotactic means, but also to facilitate their migration and proliferation. After local injection in a photothrombotic ischemic rat brain, the in situ gelation of the nanostructured hydrogel was reported, followed by significant reduction of the infarcted cavity and improved functional recovery. The authors reported enhanced endogenous neurogenesis and stimulated angiogenesis after one month of experimental treatment [124].

Highly porous and aligned hydrogel tubes based on PEG were recently proposed as platforms with in situ gelation properties for regenerative therapy in spinal cord injury. Starting from PEG microspheres and followed by an ultraviolet-initiated radical polymerization procedure performed in polydimethylsiloxane molds, PEG tubes (inner and outer diameters of 250 and 450 µm, respectively) were obtained. The emulsification-synthesized PEG microspheres (45 µm average diameter) provided suitable macroporosity (66.5%) for cellular infiltration. PEG tubes were further incorporated within a fibrin matrix, forming a 5-tube composite material that was implanted in mice with induced thoracic spinal cord injury. The composite hydrogel induced a reduced inflammatory response period and a reduced glial scar formation. Moreover, the tubular structure proved beneficial for the infiltration of endogenous cells, for the support and guidance of axon elongation, and superior oligodendrocyte-derived re-myelinization with better recovery of locomotor function [125].

Composites based on silk fibroin (SF) and biologically derived melanin were processed by electrospinning either in random or aligned nanofibrous meshes, with highly hydrophilic properties and increased thermal stability. Both melanin-containing SF materials exhibited antioxidant activity, with superior results in the case of the randomly distributed nanofibers. All composites showed superior proliferative ability for neuron-like cells, but only the aligned distribution proved more beneficial for cellular attachment and spreading. Moreover, all meshes induced neuronal differentiation of human-derived neuroblastoma cells, thus confirming the potential use of melanin-containing SF nanofibrous scaffolds for restoration and repair of CNS nerves [126].

Zeng and co-workers reported that mesenchymal stem cells (MSCs) overexpressing NT-3 receptors, differentiate into neuron-like cells and possess active roles in synapse formation following their co-culture period of 14 days alongside Schwann cells that overproduce NT-3 in 3-dimensional gelatin sponge (GS) scaffolds. When transplanted in rats with thoracic spinal cord injury, the intra-GS differentiated MSCs were partially integrated into the host neural network and promoted structural and functional hindlimb recovery [127].

More recently, the research group successfully achieved the MSC-differentiated neural network by applying previously mentioned genetic engineering and GS co-culture protocols to canine-derived bone marrow progenitor cells and sciatic nerve glial cells. The co-culture enabled the MSCs to adopt neuronal phenotypes, to develop synapse-like structures and to exhibit electrophysiological functions (as shown by assessing the presence of voltage-gated ion channels, the potential of neurotransmitter synthesis and transmission of synaptic currents). Moreover, the 3-dimensional GS scaffold provided the beneficial substrate for ECM formation. The as-developed MSC-derived neural network tissues were further transplanted in Beagle dogs with complete thoracic spinal cord transection with the long-term (6.5 months) post-transplant evaluation showing significant nerve fiber regeneration. Also, a gradual motor function recovery of the paralyzed limbs was reported both in open field and underwater locomotion tests, as well as an important recovery of the front-pelvic limb coordination and an improved electrophysiological restoration [128].

The axonal regenerative potential related to dibutyryl cyclic adenosine monophosphate (db-cAMP) and chondroitinase ABC (ChABC) was considered in the development of electrospun microfibers based on poly(propylene carbonate) (PPC) impregnated with both biomolecules. An initial burst release of db-cAMP was observed in the first 24 h, followed by a sustained release profile until day 8 and accompanied by 46.76% total agent release. A progressive release of ChABC was also observed for up to 10 days, with no burst release and with a total biomolecule release rate of 25.90%. The overexpression of neural growth-associated proteins was reported following the implantation of co-loaded PPC microfibrillar meshes in rats with hemisected thoracic spinal cords. Important axonal regenerative sprouting was also observed within the glial scars, as well as significant inhibition of glial scar formation. PPC modified microfibers provided significant motor function recovery, showing that the sustained co-delivery of db-cAMP and ChABC from polymeric matrices represents a promising strategy for CNS-related regenerative applications [129].

Another nanostructured candidate with regenerative potential for spinal cord injury treatment was proposed by considering polysialic acid (PSA) polysaccharide, whose expression level is in direct relationship with the neural cell membrane adhesion molecules, thus suggesting the possibility of modulating the intercellular and cell-ECM interactions [130,131]. Thus, Zhang and co-workers developed hybrid scaffolds based on polycaprolactone (PCL) and PSA encapsulated with methylprednisolone (MP) glucocorticoid. The electrospun nanofibrous scaffold exhibited suitable tensile strength and convenient deformability and flexibility. In the presence of astrocytes and neuroblastoma cells, the hybrid PCL/PSA/MP scaffolds were assessed as non-cytotoxic and suitable substrates for cellular attachment and proliferation. Following the implantation of PCL/PSA/MP scaffolds in rats with thoracic spinal cord lesion, the authors reported a reduced acute inflammatory response (by inhibiting local levels of pro-inflammatory cytokines) and apoptosis inhibition. The long-term tests (performed for up to 7 weeks) showed that the hybrid scaffolds significantly reduced glial scar formation, and improved axonal myelinization and superior neuronal survival, showing the improved structural and functional recovery after treatment with PCL/PSA/MP scaffolds [132].

A versatile member of the so-called smart biomedical materials and devices entity is represented by electroactive biomaterials, whose facile microstructural and electrical conductivity tailoring allows impressive possibilities for externally triggering specific bioactivities and developing genuine platforms for neuroregenerative applications [133,134,135].

Biodegradable and electrically conductive nanostructured materials were obtained by the interface polymerization of poly(3,4-ethylenedioxythiophene) nanolayer onto the channel surface of chitosan/gelatin scaffolds. The PEDOT/CS/Gel composite scaffolds, with 3-dimensional architecture and highly porous microstructure, proved suitable and superior substrates for NSCs adhesion when compared to pristine CS/Gel scaffolds, thanks to the beneficial contribution of the nanostructured PEDOT layer. Moreover, the PEDOT-assembled CS/Gel scaffolds proved enhanced cellular proliferation (as assessed by monitoring specific proliferation-related and cellular metabolism markers), maintenance of normal cell growth and superior intra-pores cellular migration. The pluripotent ability of neural stem cells cultured in the presence of PEDOT/CS/Gel scaffolds enabled their differentiation into neural cells, with superior data being reported for mature neurons and astrocytes (evaluated by up-regulation of specific proteins expression) [92].

The potential clinical implication of nanostructured biomaterials based on reduced graphene oxide (rGO) in regenerative applications for spinal cord injury was recently demonstrated. The implantation of highly porous rGO foams, with mechanical behavior similar to neural tissues, into hemisected cervical spinal cord rats did not alter the animal spontaneous behavior, nor induced toxicity in main organs. The implanted rGO foams provided structural support within the injury site and proved beneficial for infiltration and migration of endogenous cells and collagen fibers. Moreover, the rGO nanostructured aerogels determined a reduced local inflammation response, ingrowth of myelinated axons, and significant angiogenesis. rGO sheets dissociation and cellular internalization was reported at 4 months post-implantation, indicating the first biodegradability data of rGO-based materials [136].

## 4. Nanomaterials for Peripheral Nervous System Regenerative Applications

Among the smart biomaterials class—which represent the ultimate healthcare-derived desideratum in terms of specific, selective, tailored, and patient-oriented therapy—stimuli-responsive biopolymers are of great interest [137,138,139]. The physicochemical, microstructural and functional modifications occurring during certain external conditions (such as pH or temperature variation and electromagnetic or ultrasound filed presence) enable impressive tuning possibilities in developing novel, performance-enhanced and patient-oriented modern therapies [140,141,142].

Positively charged nanoparticles based on thiolated trimethyl chitosan nanoparticles grafted with tetanus neurotoxin (TMCSH-HC) and loaded with plasmid DNA encoding BDNF gene were assessed as suitable non-viral carriers for peripheral nerve injury therapy. BDNF exhibits significant actions in both CNS and PNS, being responsible for the survival and normal development of sensory and motor neurons, as well as for the promotion of growth and differentiation of new neurons and synapses [143,144,145]. Following the intramuscular administration in mice with sciatic nerve induced injury, the TMCSH-HC/BDNF platforms induced locomotor function recovery and delayed, but improved recovery of thermal and mechanical sensory functions, as well as enhancing pro-regenerative events, such as overexpression of growth-associated neural proteins, preservation of unmyelinated axons density, enhancement of myelinated axons density and protection of injury-denervated muscles [146].

Li and co-workers performed a combined micromolding—lyophilization procedure to fabricate chitosan (CS) conduits with longitudinally disposed and highly aligned ridge/groove in-wall structure and wall interpenetrated porous microstructure (porosity about 88.19 ± 0.75%). The micropatterned CS conduits exhibited mechanical behavior (tensile strength, elasticity deformation, folding and knotting flexibility) comparable to normal rat sciatic nerve. After implantation of micropatterned CS conduits in sciatic nerve injured rats, the authors reported regenerative potential comparable or slightly superior to the autografted group, including reinnervation of adjacent muscles, cellular infiltration, and intra-luminal nerve tissue growth and recovery of sciatic nerve myelination. The polysaccharide-based conduits with luminal in-wall pattern proved as promising candidates for prolonged regenerative therapy of peripheral nerves, since a slow degradation of micropatterned CS conduits was reported 3 months post-implantation [147].

Thanks to their intrinsic mechanical-induced electrical behavior, piezoelectric biomaterials attracted significant attention regarding the development of biological-mimicking structures or devices intended for particular bone tissue restorative and regenerative strategies [148,149,150,151]. Surprisingly, it was reported that calcium titanate (CaTiO_3_) nanoparticles homogenously embedded within chitosan (CS) scaffolds proved beneficial with respect to the peripheral nerve regeneration. While increasing the perovskite concentration within hybrid materials, the CS/CaTiO_3_ scaffolds exhibited decreased porosity and pore dimension and increased negative surface charge and hydrophobic feature. The addition of CaTiO_3_ nanoparticles resulted in prolonged (for up to 5 days) and beneficial effects on Schwann cells, for what concerns their normal development (as assessed following the attachment, distribution, proliferation and morpho-structural integrity of cells) and physiological behavior (as assessed by monitoring the expression of specific neural growth factors) [152].

PEI nanoparticles (40 ± 10 nm) embedded with plasmid DNA (pDNA) encoding neurotrophin-associated genes were proposed by Lackington and co-workers as suitable non-viral carriers for neural regenerative applications. The positively charged polymeric platforms, which proved superior complexation efficiency and prolonged enzymatic protection of pDNA, were assessed as enhancers for Schwann cell transfection efficiency (60 ± 13%), with no long-time effects in terms of proliferation and metabolic activity. The polymer complexes loaded with genes encoding NGF, GDNF (glial derived neurotrophic factor, responsible for the survival of motor and sensory neurons and Schwann cells) [153,154] and c-Jun (transcription factor implied in reprogramming and up-regulation of injured Schwann cells) [155,156], proved superior in expressing neurotrophic cytokines and promoting neurite outgrowth in both Schwann and neuronal cells derived from rat dorsal root. The most promising cellular regenerative potential was assigned to the PEI-pDNA nanosystems embedded with c-Jun encoding gene [157].

Zhang and co-workers proved that PLGA microspheres represent a promising platform for sustained and sequential delivery of erythropoietin (EPO) and NGF. To avoid early nerve apoptosis following the administration of NGF, the protein release was postponed by introducing bovine serum albumin (BSA) within the PLGA microsphere matrix. Motor function recovery, electrophysiological improvement, fibrosis decrease, and axon myelinization increase were reported at 2 months after the co-administration of EPO/PLGA and NGF/BSA-PLGA microsystems in sciatic nerve injured rats [158].

The immobilization or incorporation of nectin-like molecule 1 (NECL1) onto or within biomaterials represents an attractive strategy to mimic the microenvironment of the nervous system. NECL1 is an immunoglobulin-like molecule specifically expressed by the neural tissue, being responsible for cell-cell interactions, synapse and myelinated axons formation and brain morphogenesis [159,160,161]. PLGA membranes modified with NECL1 (50 ng/mL concentration) proved as beneficial substrates for the attachment, proliferation and normal growth of rat-derived Schwann cells (as shown by both morphological consideration and neurotrophic factor gene expression). Furthermore, the composite biomaterials were mechanically processed to form PLGA/NECL1 conduits which were implanted in rats with induced sciatic nerve injury. Following a 3-month animal treatment, progressive time-dependent events were reported, such as significant functional recovery of sciatic nerve, improved recovery of nerve conduction velocity and reduced weight loss of adjacent muscle. Since both motor and sensory nerve functions were restored and the local non-aberrant reinnervation was promoted, the PLGA/NECL1 conduits were proposed as promising candidates for peripheral nerve regenerative applications [162].

Artificial conduits based on linearly oriented multi-walled silk fibroin / silk sericin blend (SF/SS, 90/10 wt.%) coated with a hollow PLGA sheath were successfully assessed for peripheral nerve regenerative applications. At 45 days after implantation in rats with sciatic nerve defects, the highly transparent and porous PLGA/SF/SS conduits showed no clear signs of systemic or local inflammation, but determined the gradual recovery of limb motor function. In comparison with the autografted rats, the PLGA/SF/SS-treated animals manifested similar electrophysiological recovery, slightly superior regenerated nerve fibers and myelinated nerve axons and comparable adjacent muscle reinnervation [163].

The beneficial implications of electrically active biomaterials in neural tissue regeneration [134,164] enabled the evaluation of highly porous and hydrophobic meshes based on poly(L-lactic) acid (PLLA) for such specific applications. The PLLA meshes, consisting of highly aligned polymeric nanofibers obtained by electrospinning, proved a narrow distribution of thermally stimulated depolarization currents and a high stability of corona-induced polarization (for up to 6 months). The developed nanostructured materials were evaluated as suitable platforms for the proliferation of neuroblastoma cells, while their intrinsic polarization potentiated the neuronal differentiation under retinoic acid exposure. Moreover, as assessed on primary embryonic cortical neuronal cultures, the presence of polarized PLLA meshes (either positively or negatively poled surfaces) stimulated an improved neuritogenesis with ~30%, in comparison with non-poled meshes [165]. Also, PLLA nanofibrous meshes coated with polypyrrole (Ppy) proved to induce the transdifferentiation of bone mesenchymal stromal cells (BMSCs) into neural cells in the presence of external electrical stimulation. Moreover, the addition of NGF to the culture medium or the modification of PLLA/Ppy nanofibers with laminin proved synergistic effects with respect to the transdifferentiation ability of BMSCs under proper electrical stimulation conditions [166].

Jing and co-workers proposed composite conduits based on electrospun parallel-aligned PLGA fiber-based meshes coated with Ppy for peripheral nerve regeneration. When compared to electrospun PLGA mesh, the PLGA/Ppy fibrous mesh proved good dimensional stability following its testing under physiologically simulated conditions, but slightly reduced tensile strength and elasticity. On the other hand, when assessed in the presence of primary neuron-like cells, the Ppy-coated PLGA fibrous meshes displayed non-cytotoxicity, enhanced cellular attachment, fast cellular proliferation rate, and predominant oriented cellular morphology. After inductive culture conditions, the PLGA/Ppy composite also induced the most significant neurite outgrow and spreading, indicating the potential of parallel-aligned conductive fibers for mature neural cells differentiation. The 12-week evaluation performed under physiological conditions showed that the constructed PLGA/Ppy conduit possessed prolonged and sustained degradation (accompanied by ~40% weight loss) and maintained their tubular morphology. Following the implantation of PLGA/Ppy fibrous conduits in sciatic nerve transected rats and compared to autografted animals, similar functional and electrophysiological recovery and regenerated axons were reported 12 weeks after implantation procedure [167].

The non-cytotoxic and superior electroactivity related to Ppy was also employed to modify the surface of poly(L-lactide-co-ε-caprolactone)/silk fibroin (PLCL/SF) nanofibrous membranes. Polypyrrole nanoparticles were superficially deposited onto electrospun nanofibers by applying an oxidative polymerization process. In comparison with pristine PLCL/SF membranes, the Ppy-coated composites exhibited similar thermal stability, but possessed electrical conductivity and pronounced hydrophilicity, as well as enhanced mechanical properties. The PLCL/SF-Ppy membranes proved beneficial for the proliferation of Schwann cells under normal culture conditions, but an external electrical stimulation encouraged even more enhanced proliferation rates and spreading. Superior cellular proliferation was also reported in the case of primary neuron-like cells cultured with PLCL/SF-Ppy nanofibrous membranes. However, only by applying electrical stimulation, the differentiation ability of neuron-like cells into mature neural cells was reported, as demonstrated both by the presence of many branched neurites and extended axons and by the monitoring of neuron-associated gene expression [168]. By the facile modification of the collector during the electrospinning process, the same research group fabricated hollow conduits of PLCL/SF, whose surface was further modified with a Ppy nanoparticle layer. After implantation of PLCL/SF-Ppy conduits in rats with sciatic nerve injury, the authors reported re-bridged and regenerated nerves. In comparison with autografted subjects, the PLCL/SF-Ppy-treated animals possessed similar local invasion and proliferation of endogenous Schwann cells in both early and later healing stages (4 and 12 weeks post-surgery, respectively), but similar promotion of myelin formation only in later post-surgical stage. The long-term microstructural investigations showed that the PLCL/SF-Ppy conduits enabled the substitution of the induced nerve gap with regenerated nerve tissue, which mainly consisted of myelinated nerve fibers. Moreover, the functional recovery of the PLCL/SF-Ppy-treated animals was comparable to the autografted ones, showing that the polypyrrole-coated PLCL/SF nanofibrous conduits could represent promising alternatives for autograft-based therapy in peripheral nerve regeneration [169].

By combining molding and thermally induced phase separation procedures, Uz and co-workers developed gelatin-based conduits for MSC cultures. The synthesized platforms possessed different microstructures, namely nanofibrous (NF, opaque after water contact, total porosity of ~95%, swelling ratio of ~160%, the stiffest conduit), macroporous (MP, transparent after water contact, pore size of ~100 µm, swelling ratio of ~800%) or ladder-like (LL, transparent after water contact, pore size of ~150 µm, swelling ratio of ~900%), but preserved their elastic behavior and maintained their solid nature at physiological temperatures. The cultivation of MSCs in the presence of gelatin conduits demonstrated improved cellular proliferation, but emphasized increased cellular spreading, cellular network interconnection, and superior long-term cellular survival only for MP and LL structures. Both MP and LL gelatin conduits proved high efficiency for the direct transdifferentiation into Schwann cells, as shown by monitoring specific markers of glial cells. Still, the most promising data was attributed to the LL gelatin conduits, which further showed the highest neurotrophic factors secretory capacity and the greatest growth of neurite extension (the last being assessed in a co-culture experiment). The proposed 3-dimensional conduits based on natural-derived gelatin are promising platforms for MSCs-based regenerative therapy of peripheral nervous tissue [170].

Another study emphasized the potential use of fibrous scaffolds based on highly aligned electrospun polycarbonate urethane (PCU) modified with neuroscience-relevant cationic hydrophilic biomolecules of Poly-L-Lysine (PLL) or Poly-L-Ornithine (PLO). When compared to pristine PCU scaffolds, both PLL- and PLO-modified materials significantly improved cellular attachment and subsequent cellular proliferation in dental pulp stem cells cultures (with intrinsic potential for neuronal differentiation). The most promising results, in terms of peripheral nerve regeneration potential, were assigned to the PLO-modified PCU scaffolds [171].

Particular representatives of electroactive materials with tremendous implications in neuroregenerative applications are graphene and its derivatives (that can be obtained by applying various physical or chemical protocols). The atomic structure and electron distribution related to these materials mainly dictate their superior properties, such as excellent mechanical behavior, distinctive optical properties, increased thermal stability, superior electrical conductivity, excellent chemical stability [172,173,174].

Single layers of hydrophilic graphene oxide (GO) and hydrophobic reduced graphene oxide (rGO) proved as beneficial substrates for neuronal differentiation of adipose-derived stem cells (ADSCs), with a neurogenic differentiation rate above 90%. In particular, GO-based mats provide an enhanced proliferation and differentiation rate of ADSCs into neuron-like cells [175].

Wang and co-workers proposed the combination of allogeneic decellularized scaffolds and GO for regeneration of injured peripheral nerve. Following the implantation procedure in rats with sciatic nerve trauma, neither acute inflammation nor mid-term toxic effects (as shown by monitoring the level of hepatic enzymes) were reported. Starting from the 11th week after implantation, the authors reported muscle strength rehabilitation and electrophysiological functional recovery [176].

Electrospinning fabricated hybrid scaffolds based on sodium alginate (SA) and polyvinyl alcohol (PVA) embedded with graphene nanosheets proved suitable microstructural features (uniform and interconnected porous structure with superior hydrophobicity and prolonged degradation rate), as well as beneficial mechanical (simultaneously improved tensile strength, toughness and elongation) and electrical properties for peripheral nerve regeneration [177].

Reduced graphene oxide (rGO) nanosheets were deposited onto the surface of composite SF/PLCL electrospun fibers towards the fabrication of scaffolds with randomly oriented constituent nanofibers, nanoporosity and nanoroughness, prominent hydrophobic behavior, enhanced mechanical properties, and prolonged conductive stability. The SF/PLCL/rGO scaffolds proved beneficial effects regarding the proliferation, spreading, myelination, and neurotrophin secretion of Schwann cells, the results being superior under electric stimulation conditions. The neurotrophin-enriched medium resulted by removing the Schwann cells cultured with SF/PLCL/rGO proved enhanced differentiation of the primary neuron-like cells. Moreover, the polymer scaffolds coated with graphene derivative showed differentiation of neuron-like cells, with superior results after electrical stimulation. After the implantation of SF/PLCL/rGO conduits into sciatic nerve injured rats, significant locomotor and electrophysiological functional recovery of the regenerated nerve was reported (as assessed both at 4 and 12 weeks post-surgery and compared with autografted animals). Also, the fabricated composite conduits showed no local inflammation but important invasion and proliferation of endogenous Schwann cells and induced significant nerve fiber regeneration and re-myelinization. Given the fact that the healing capacity exhibited under endogenous electrical stimulation (animal model) by the graphene-derivative-modified SF/PLCL conduits was similar to autografts, the SF/PLCL/rGO scaffolds possess impressive potential for peripheral nerve repair and regeneration [178].

## 5. Conclusions

The complex microstructure and ultrastructure and the intricate physiology and pathophysiology of the nervous tissue represent major challenges in developing new therapeutic strategies. Autografts and stem cells—based therapies represent the current standards in nervous tissue regenerative strategies. Even if substantial efforts have been oriented towards intensive research, the treatment possibilities with clinically acknowledged efficiency for minimizing or reversing the damaged nervous system are currently limited.

However, the impressive progress in nanotechnology with regards to molecular biology, biotechnology, biophysics, biochemistry, materials science, and bioengineering enables researchers to allocate resources towards specific, specialized, and personalized therapeutic approaches for the nervous tissue. In the particular case of injured CNS and PNS, modern regenerative strategies focus on exploring the tunable biological multifunctionality related to novel biocompatible nanomaterials.

Nanopharmaceuticals and nanostructured particles, nanostructured films and membranes, nanostructured gels, and scaffolds showed promising clinical potential for neuroregeneration, as efficient platforms for loading and conditional release of neuroprotective drugs, growth factors, neurotrophins, chemokines, genes, and stem cells relevant in both CNS and PNS therapy. As discussed above, biocompatible nanomaterials represent ideal candidates for the future of performance-enhanced strategies in the field of nervous system repair and regeneration.

## Abbreviations

ADSCs	adipose-derived stem cells
BBB	blood-brain barrier
BDNF	brain-derived neurotrophic factor
bFGF	basic fibroblast growth factor
BMSCs	bone mesenchymal stromal cells
BSA	bovine serum albumin
ChABC	chondroitinase ABC
CNS	central nervous system
cRGD	cyclic arginine-glycine-aspartate
CS	chitosan
db-cAMP	dibutyryl cyclic adenosine monophosphate
DNA	deoxyribonucleic acid
ECM	extracellular matrix
EGF	epidermal growth factor
EPO	erythropoietin
GDNF	glial derived neurotrophic factor
Gel	gelatin
GFs	growth factors
GO	graphene oxide
GS	gelatin sponge
HA	hyaluronic acid
HA-Lm	hyaluronic acid-laminin
HAMC	hyaluronic acid-methylcellulose
IMPs	immune-modifying nanoparticles
LL	ladder-like
MMP	matrix metalloproteinase
mNSCs	mouse-derived neural stem cells
MP	macroporous
MP	methylprednisolone
MSCs	mesenchymal stem cells
NECL1	nectin-like molecule 1
NF	nanofibrous
NGF	nerve growth factor
NPSCs	neural progenitor stem cells
NSCs	neural stem cells
NT-3	neurotrophin-3
PCL	polycaprolactone
PCN	polyelectrolyte complex nanoparticles
PCU	polycarbonate urethane
pDNA	plasmid DNA
PEDOT	poly(3,4-ethylenedioxythiophene)
PEG	polyethylene glycol
PEI	polyethyleneimine
PLCL	poly(l-lactide-*co*-ε-caprolactone)
PLGA	poly(lactide-*co*-glycolide)
PLL	poly-l-Lysine
PLLA	poly(l-lactic) acid
PLO	poly-l-Ornithine
PNS	peripheral nervous system
PPC	poly(propylene carbonate)
Ppy	polypyrrole
PSA	polysialic acid
PVA	polyvinyl alcohol
rGO	reduced graphene oxide
SA	sodium alginate
SDF-1	stromal cell-derived factor 1
SF	silk fibroin
SS	silk sericin
TAT	twin-arginine translocation
TMCSH-HC	thiolated trimethyl chitosan nanoparticles grafted with tetanus neurotoxin
XMC	cross-linked methylcellulose
